# Revisiting Hyaluronan Catabolism in *Bacteroides*: Pathway Conservation, Overlooked Proteins, and Predictive Accuracy

**DOI:** 10.1002/mbo3.70227

**Published:** 2026-02-05

**Authors:** Martin Sindelar, Anna Kocurkova, Matej Simek, Pavel Roudnicky, Gabriela Ambrozova, Lukas Kubala, Kristyna Turkova

**Affiliations:** ^1^ Institute of Biophysics of the Czech Academy of Sciences, Department of Biophysics of Immune System Brno Czech Republic; ^2^ Faculty of Science Institute of Experimental Biology Masaryk University Brno Czech Republic; ^3^ Contipro a.s. Dolní Dobrouč Czech Republic; ^4^ Central European Institute of Technology (CEITEC), Proteomics Core Facility Masaryk University Brno Czech Republic

**Keywords:** *Bacteroides*, comparative proteomics, glycosaminoglycans, hyaluronan, polysaccharide utilization loci

## Abstract

The ability of gut microbes to degrade host‐ and diet‐derived glycans is central to microbiome ecology and host interactions, yet predicting these functions in silico remains challenging. Hyaluronan (HA), a glycosaminoglycan (GAG) abundant in host tissues and dietary supplements, is depolymerized by specialized polysaccharide utilization loci (PULs) in *Bacteroides*. Here, we combined comparative protein analysis, functional assays, and quantitative proteomics to evaluate the reliability of sequence‐based predictions of HA utilization. Clustering of more than 3900 PL8 and GH88 protein sequences from 54 *Bacteroides* species did not distinguish known HA degraders from nondegraders, underscoring the limited predictive power of these enzymes alone. Experimental validation in *Bacteroides acidifaciens* DSM 111135 and *Bacteroides thetaiotaomicron* DSM 2079 confirmed HA degradation, as HA‐derived fragments were identified by liquid chromatography–mass spectrometry. Proteomic profiling revealed coordinated induction of both canonical GAG‐specific PULs‐encoded proteins and noncanonical accessory proteins (BT4410/BT4411) in response to HA in both species. Incorporating such noncanonical components into comparative frameworks may improve prediction of glycan utilization potential and help link microbial genomic content to ecological function in the gut.

## Graphical abstract

Hyaluronan degradation in *Bacteroides* cannot be reliably predicted from canonical PL8/GH88 enzymes alone. Experimental validation reveals coordinated induction of noncanonical accessory proteins (BT4410/BT4411), highlighting pathway plasticity and context‐dependent glycosaminoglycan utilization.

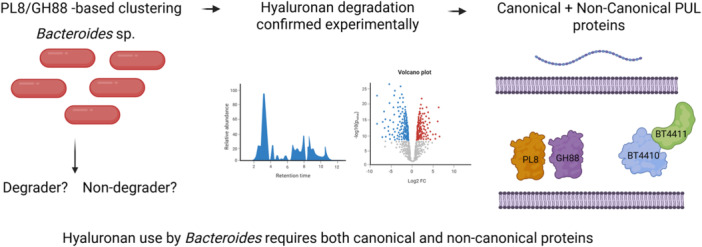

## 1 Introduction

The mammalian gastrointestinal tract (GIT) harbors a dense and taxonomically diverse microbial community that plays a crucial role in maintaining host physiological homeostasis, including metabolic function, immune modulation, and barrier integrity (Maciel‐Fiuza et al. 2023; Takiishi et al. 2017). The specific taxonomic composition of the gut microbiota is highly dynamic and shaped by a variety of host‐ and environment‐related factors, such as genetic background, age (Schoultz et al. 2025), microbial exposure, and most notably, dietary inputs (Ross et al. 2024; Zmora et al. 2019). In addition to diet, host‐derived glycans decorating the intestinal mucosa constitute a major ecological determinant that shapes microbial colonization, metabolism, and host–microbiota interactions, while simultaneously contributing to immune regulation and barrier integrity through the mucus–epithelial interface (Crouch et al. 2024; Demirturk et al. 2024).

Within this ecosystem, the genus *Bacteroides*, dominating across a wide range of mammalian hosts (Mariat et al. 2009), represents a functionally versatile and ecologically prominent component of the GIT (Shin et al. 2024), largely owing to its exceptional capacity to degrade structurally complex dietary polysaccharides (Rawat et al. 2022). This capacity is largely attributed to the presence of polysaccharide utilization loci (PULs), modular genomic regions that encode a coordinated set of proteins involved in the recognition, binding, depolymerization, and uptake of diverse glycans (Terrapon et al. 2018; H. Liu et al. 2021). The specificity and variability of PULs within *Bacteroides* genus enable adaptation to distinct polysaccharide substrates, contributing significantly to their ecological success and functional specialization within the gut environment (Raghavan and Groisman 2015). Importantly, this versatility also extends to the utilization of structurally diverse host‐associated glycans, further expanding the substrate spectrum encoded by PULs (Crouch et al. 2024; Demirturk et al. 2024).

To explore the substrate‐specific diversification of different bacteria, several in silico approaches have been proposed. Notably, Kappelmann et al. (2019) demonstrated that bacteria can be clustered based on the composition of their SusC/D‐like (starch utilization system) transporter complexes, key PUL‐encoded proteins, which facilitate substrate‐specific glycan import into the bacterial periplasm (Kappelmann et al. 2019). While SusC/D‐based clustering provides useful resolution for structurally diverse glycans, a more detailed analysis is required when investigating structurally and functionally closely related glycans, such as glycosaminoglycans (GAGs).

GAGs, including chondroitin sulfate, heparan sulfate, and hyaluronan (HA), represent a class of diet‐associated polysaccharides that are metabolically relevant to gut bacteria. The ability of certain *Bacteroides* species to utilize GAGs as nutrient sources provides a significant ecological advantage, facilitating their expansion in the gut microbial community. Supporting this, oral administration of HA has been shown to selectively enrich genus *Bacteroides* in vivo (Šimek et al. 2023 *)*. While mammalian tissues express hyaluronidases, these enzymes differ substantially from their bacterial counterparts in structure and catalytic mechanism (Sindelar et al. 2021). Moreover, accumulating evidence demonstrates that the primary degradation of HA within the GIT is mediated by the gut microbiota rather than by host‐derived enzymes (Šimek et al. 2023). This highlights the ecological importance of microbial enzymes and motivates a deeper investigation into their enzymatic diversity and specificity.

Mechanistic insights into GAG degradation by *Bacteroides* have been established through the characterization of specialized GAG‐specific PUL. A study by Ndeh (2020) comprehensively described GAG‐specific PUL in *Bacteroides thetaiotaomicron*, which has since served as a model system for dissecting the molecular basis of GAG catabolism (Ndeh 2020). Building upon this, Overbeeke et al. (2022) investigated homologous GAG‐specific PUL genes in a broader panel of *Bacteroides* species, demonstrating that the ability to degrade GAGs is not universally conserved but instead exhibits considerable interspecies variability (Overbeeke et al. 2022). While many studies classify *Bacteroides* species with a binary “degrader” or “non‐degrader” label, the underlying genetic and functional diversity suggests that HA utilization may be a quantitative trait. This raises the possibility that sequence conservation alone may be insufficient to explain functional variation. Recent studies further confirm that *Bacteroides* species vary widely in their GAG‐degrading capacities, ranging from non‐degraders to strains capable of utilizing multiple GAG substrates, including HA, and chondroitin sulfate (Akazawa et al. 2023; Fang et al. 2024; Kawai et al. 2018).

The GAG degradation pathway in *B. thetaiotaomicron* has been extensively characterized, providing a detailed biochemical and genetic framework for understanding GAG metabolism. Key enzymatic components of this pathway include BT3324 and BT3350, members of the polysaccharide lyase family 8 (PL8), and BT3348, a representative of glycoside hydrolase family 88 (GH88). These enzymes exhibit catalytic activity toward GAG‐derived substrates and are well annotated in public databases (NCBI, Uniprot), making them suitable candidates for comparative analyses and pathway‐based clustering. However, while PL8 and GH88 enzymes are functionally relevant for studying GAG degradation in general, they lack the substrate specificity required to distinguish between individual GAGs, such as chondroitin sulfate and HA (Overbeeke et al. 2022). In contrast, BT4410, a member of polysaccharide lyase family 33 (PL33), has been suggested to possess higher specificity for HA degradation (Pan et al. 2021; Yin et al. 2025). Despite this specificity, the limited representation and annotation of PL33 homologs in public databases currently constrain its applicability in comparative studies (Drula et al. 2022). Consequently, PL8 and GH88 enzymes remain the most practical targets for pathway‐level clustering due to their broader distribution and consistent annotation across *Bacteroides* genomes.

In this study, we hypothesize that *Bacteroides* species can be grouped according to their enzymatic potential to degrade GAGs, as inferred from the presence and sequence conservation of key PL8 and GH88 family enzymes, together with comparative analyses of proteins encoded by GAG‐specific PUL. We further propose that such clustering reflects shared metabolic capacities among functionally related species and provides insights into the distribution and substrate specificity of GAG‐degrading phenotypes. To address this, we combined in silico comparative analyses with targeted cultivation experiments on HA, a representative GAG, followed by proteomic profiling of GAG‐specific PUL‐encoded proteins. This combined approach aims to identify functional signatures linked to HA utilization, uncover previously unrecognized HA‐degrading capacities, and enable prediction of metabolic potential in *Bacteroides* species currently lacking comprehensive characterization.

## 2 Results and Discussion

### 2.1 Comparative Clustering of PL8 and GH88 Enzymes in *Bacteroides* Reveals Limited Predictive Power for HA‐Degrading Phenotypes

**Figure 1:**
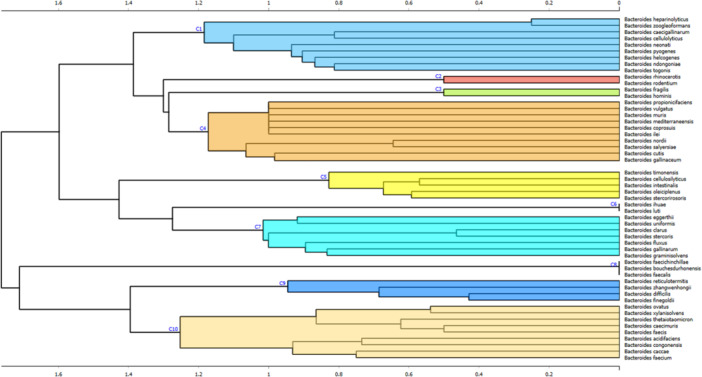
Hierarchical clustering of *Bacteroides* species based on sequence identity of two GAG‐specific PUL‐encoded enzymes, polysaccharide lyase family 8 (PL8) and glycoside hydrolase family 88 (GH88). The analysis included 54 *Bacteroides* species. Hierarchical clustering was performed in Orange Data Mining software using a distance matrix constructed from the presence or absence of PL8 and GH88 sequence clusters, with distances calculated using the Jaccard metric. The horizontal axis of the dendrogram represents the clustering distance derived from this distance matrix, reflecting dissimilarity in PL8/GH88 enzyme cluster composition between species. The resulting dendrogram displays 10 distinct clusters (C1–10). GAG, glycosaminoglycan; PUL, polysaccharide utilization locus.

Clustering of two key GAG‐degrading enzyme families, PL8 and GH88, was performed using 1921 PL8 and 2051 GH88 protein sequences retrieved from UniProt and NCBI. Phylogenetic analysis grouped 54 *Bacteroides* species into 10 clusters (C1–C10) (Figure 1). Cluster sizes ranged from 2 to 11 species, with C1, C4, C7, and C10 being the largest, while C2, C3, C6, and C8 contained only a few representatives. Full species composition is shown in Figure 1.

**Figure 2:**
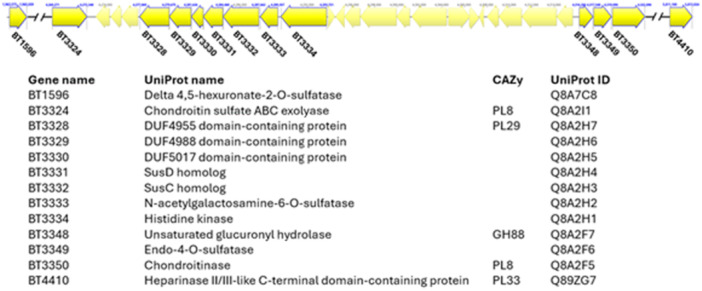
Genomic organization of the GAG‐specific PUL in *Bacteroides thetaiotaomicron*. Diagram constructed based on protein annotations and locus architecture curated in UniProt (Bateman et al. 2021). Visualized using Geneious Prime (Geneious Prime 2024.0.5). GAG, glycosaminoglycan; GH88, glycoside hydrolase family 88; PL8, polysaccharide lyase family 8; PUL, polysaccharide utilization locus.

To evaluate the conservation of GAG‐specific PUL‐encoded proteins, we performed BLASTp homology searches across all 10 clusters, using the GAG‐specific PUL‐encoded proteins from *B. thetaiotaomicron* as the reference set (Ndeh 2020) (Figure 2). GAG‐specific PUL‐encoded proteins include surface‐associated polysaccharide lyases (BT3328, PL29, and UniprotID Q8A2H7), glycan‐binding proteins (BT3329, UniprotID Q8A2H6; BT3330 UniprotID Q8A2H5), SusC/D‐like transporters (BT3331, UniprotID Q8A2H4; BT3332, UniprotID Q8A2H3), periplasmic lyases (BT3324, PL8, UniprotID Q8A2I1; BT3350, PL8, UniprotID Q8A2F5), Heparinase II/III‐like C‐terminal domain‐containing lyase (BT4410, PL33, and UniprotID Q89ZG7), a glycoside hydrolase active on unsaturated disaccharides (BT3348, GH88, and UniprotID Q8A2F7), sulfatases (BT3333, UniprotID Q8A2H2; BT3349, UniprotID Q8A2F6; BT1596, UniprotID Q8A7C8), and a histidine kinase (BT3334, hybrid two‐component sensor, UniprotID Q8A2H1) (Figure 2). All these proteins form a spatially coordinated canonical catabolic system for GAG degradation, including HA and chondroitin sulfate. Recent syntheses of gut microbial GAG metabolism show that GAG utilization in *Bacteroides* depends on a broader and more flexible enzymatic repertoire than is captured by individual enzyme families alone. In addition to PL8 and GH88, multiple polysaccharide lyase families, diverse sulfatases, and alternative transport systems contribute to GAG depolymerization and uptake in lineage‐specific combinations organized within highly variable PULs. As a result, conserved pathway logic coexists with substantial divergence in individual enzymes and accessory proteins, allowing functionally equivalent HA‐degrading phenotypes to arise from distinct molecular configurations and limiting phenotype inference from a small subset of CAZyme families (Dong et al. 2024).

Within cluster 10 (C10), GAG‐specific PUL‐encoded protein homologs were generally well conserved, showing sequence identity above 60%. However, the surface‐associated components BT3328–BT3330 consistently exhibited the lowest conservation, with identity dropping to only 30%–40% in some species. This uneven conservation suggests that substrate capture rather than downstream processing may represent a major axis of functional divergence, consistent with prior work showing that glycan specificity in *Bacteroides* is largely dictated by surface binding and transport components rather than core catabolic enzymes (Zafar and Saier 2021).

BT3328, a surface polysaccharide lyase implicated in the initial cleavage and uptake of long‐chain HA (Overbeeke et al. 2022; Ndeh et al. 2018), displayed only ~40% amino acid identity to the *B. thetaiotaomicron* ortholog. The particularly low conservation of surface‐associated components is notable in light of their ecological role. Outer membrane glycan‐binding proteins in *Bacteroides* are exposed to highly variable host‐ and diet‐derived glycans and are therefore subject to strong diversifying selection (Zafar and Saier 2021). The value 40% lies at the lower threshold for reliable functional annotation (Kroll et al. 2023), and functional conservation is known to decline sharply below this level (Tian and Skolnick 2003). Nevertheless, previous experiments targeting BT3328 have confirmed HA‐degrading activity (Ndeh et al. 2018) and highlighted its importance in HA catabolism in *Bacteroides* spp. (Overbeeke et al. 2022). In line with these observations, the literature indicates that many bacterial species from the C10 group are capable of degrading HA (Table S1), suggesting that, despite low sequence identity, BT3328 or alternative functionally analogous enzymes may retain activity toward HA.

In other clusters, inconsistencies emerge between the homology of GAG‐specific PUL‐encoded proteins and the observed phenotype (Tables S1 and S3). For example, *Bacteroides finegoldii* possesses GAG‐specific PUL‐encoded proteins highly similar to those of *B. thetaiotaomicron* and has been experimentally confirmed to degrade HA (Overbeeke et al. 2022; Akazawa et al. 2023; Fang et al. 2024), yet it is assigned to a different cluster (C9) than most HA degraders. Conversely, *Bacteroides fragilis* displays a comparable GAG‐specific PUL‐encoded proteins profile but is consistently reported as an HA nondegrader (Ndeh 2020; Overbeeke et al. 2022; Fang et al. 2024). Together, these cases demonstrate that even strong conservation of canonical GAG–PUL components is insufficient to predict HA utilization, highlighting fundamental limits of PL8/GH88‐based clustering. This disconnect is consistent with the ecological plasticity of *Bacteroides*, in which glycan utilization depends on coordinated regulation, transport capacity, and environmental context rather than enzyme presence alone (Rawat et al. 2022; Zafar and Saier 2021). Quantitatively, agreement between cluster assignments and experimentally verified HA‐cleaving labels was modest (ARI = 0.297; NMI = 0.299). Majority‐vote mapping on the labeled subset gave precision = 1.00, recall = 0.80, *F*1 = 0.889, and accuracy = 0.895. Clusters C9 and C10 aligned with HA cleavers; C1, C3, C5, and C7 with noncleavers; C4 was split; C2, C6, and C8 had no labeled taxa. Most taxa (34/53) were Mixed/NA and excluded from metrics. Overall, clustering captured only a weak signal: perfect precision suggests the specificity of predicted cleavers, but reduced recall and mixed C4 show limited coverage. This likely reflects biological heterogeneity (conditional HA use and variation beyond PL8/GH88) and methodological limits (small labeled set). Thus, cluster membership should be seen as a tentative indicator, not sufficient to classify species as HA cleavers.

This inconsistency is further evident in C4 and C7, which contain a mix of reported HA degraders, confirmed HA nondegraders, and species with untested HA‐degrading potential (Table S1). Such mixed clusters are consistent with the view that HA utilization in *Bacteroides* represents a quantitative, context‐dependent trait shaped by regulation, substrate availability, and microbial interactions rather than a binary degrader/nondegrader phenotype (Zafar and Saier 2021). Notably, *B. salyersiae* demonstrates robust HA‐degrading activity despite lacking protein homologs for BT3328 and its associated binding proteins BT3329 and BT3330. This suggests the use of an alternative mechanism, possibly involving functionally analogous but structurally divergent proteins for HA capture and cleavage (Fang et al. 2024). The absence of detectable homology to BT4410, an enzyme with high HA affinity (H. Liu et al. 2021), further supports the existence of multiple, nonorthologous enzymatic strategies for HA degradation within the genus, consistent with broader observations of functional convergence in *Bacteroides* glycan utilization systems (Zafar and Saier 2021).

Taken together, PL8/GH88‐based clustering reveals that HA utilization in *Bacteroides* is a quantitative, context‐dependent phenotype shaped by pathway plasticity, regulation, and ecological pressures, and therefore cannot be reliably inferred from sequence similarity alone (Rawat et al. 2022; Dong et al. 2024). This limitation motivated experimental validation in *Bacteroides acidifaciens*, a C10 species that clusters with established HA degraders but whose GAG‐specific PUL components have not been functionally characterized. Independent in vivo studies further support the ecological relevance of *B. acidifaciens*, as chondroitin sulfate disaccharide supplementation markedly increased its abundance and altered pathways linked to GAG metabolism and regulation, even though HA degradation itself was not directly assessed (F. Liu et al. 2017).

### 2.2 Intraspecies Variability Among *B. acidifaciens* Strains

To assess potential strain‐level variation in GAG‐specific PUL‐encoded proteins of *B. acidifaciens*, we compared 226 available proteomes to the GAG‐specific PUL‐encoded proteins of *B. thetaiotaomicron*, used as the reference set (Ndeh 2020). The presence/absence profiling revealed near‐universal conservation for 11 of the 13 proteins, protein homologs were detected in > 98% of strains (Table S2). Sequence identity across strains was also exceptionally high, with mean amino acid identities ranging from 97.2% (BT3331) to 99.7% (BT3328) and a median standard deviation of 0.6 percentage points. Most proteins showed minimal divergence (SD < 1%), suggesting strong purifying selection on the pathway. Two components BT3331 (SusC‐like transporter) and BT3348 (GH88 glycoside hydrolase) displayed higher variability (SD > 5 pp), which could reflect localized adaptation, horizontal gene transfer, or lineage‐specific diversification. Transporter proteins such as SusC/SusD are known to exhibit sequence plasticity in *Bacteroides*, enabling adaptation to host‐ or niche‐specific glycans (Martens et al. 2009; Grondin et al. 2017). GH88 enzymes (e.g., BT3348), which act on unsaturated disaccharides, have also been reported to show functional diversification associated with substrate range variation (Cartmell et al. 2017).

Taking together, these findings indicate that GAG‐specific PUL‐encoded proteins are highly preserved within *B. acidifaciens* and are unlikely to exhibit strain‐specific variation. This justified the selection of *B. acidifaciens* DSM 111135 as a representative model for downstream functional validation of GAG‐specific PUL‐encoded proteins using HA as substrate.

### 2.3 HA‐Degrading Ability and GAG‐Specific PUL‐Encoded Proteins Expression in *B. acidifaciens*

Cluster C10 is enriched in *Bacteroides* species that shows high identity to the *B. thetaiotaomicron* GAG‐specific PUL‐encoded proteins and reported to degrade HA (Tables S1 and S3), which would be expected to predict HA utilization. However, as shown above, PL8/GH88‐based clustering does not reliably group *Bacteroides* species by HA‐degrading phenotype, and GAG‐specific PUL‐encoded proteins identity alone does not guarantee functional activity (HA utilization). To address this uncertainty, we experimentally assessed the HA‐degrading capacity of *B. acidifaciens* DSM 111135, a C10 member, and compared its GAG‐specific PUL‐encoded proteins expression and HA degradation ability with those of the model HA degrader *B. thetaiotaomicron* DSM 2079.

**Figure 3:**
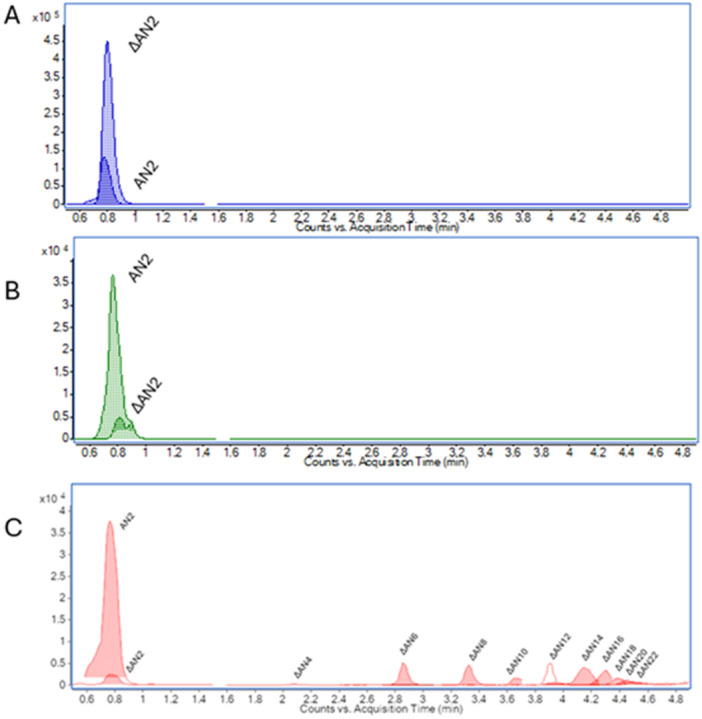
Analysis of degradation products after cultivation of bacterial strains in hyaluronan‐based minimal medium using liquid chromatography–mass spectrometry: (A) *Bacteroides thetaiotaomicron*, (B) *Pedobacter heparinus*, and (C) *Bacteroides acidifaciens*. AN2, saturated disaccharides; ΔAN2–ΔAN22, unsaturated oligosaccharides ranging from 2 (ΔAN2) to 22 (ΔAN22) subunits.

Both species were cultivated in standard nutrient‐rich media and in HA‐based minimal medium to reach the stationary phase of growth (Figure S1). In both cases, growth was sustained, but cultures in HA‐based minimal medium grew markedly slower and reached lower final optical densities than in rich medium. Doubling times in HA‐based medium were approximately twice as long, consistent with previous reports that growth on HA as the sole carbon source is slower due to the complexity of polymer breakdown and transport (Overbeeke et al. 2022). To monitor HA degradation during growth, culture supernatants from both *Bacteroides* strains were analyzed alongside *Pedobacter heparinus* DSM 2366, a well‐characterized HA degrader used as a positive control (Kawai et al. 2018; Hashimoto et al. 2014). Degradation products were profiled using agarose gel electrophoresis (Figure S2) and liquid chromatography–mass spectrometry (LC–MS) (Figure 3).

In *B. thetaiotaomicron* culture medium, HA was degraded primarily into unsaturated disaccharides (ΔAN2) (Figure 3A). These low‐molecular‐weight fragments were undetectable by agarose gel electrophoresis (Figure S2, lanes 5–7) but were clearly identified by LC–MS, in agreement with the known sensitivity differences between methods (Perez et al. 2023). The degradation profile closely matched that of *P. heparinus* (Figure 3B; Figure S2, lane 1), confirming rapid and complete depolymerization of HA into low‐molecular‐weight products. In contrast, *B. acidifaciens* produced a broader spectrum of degradation products, including both disaccharides and longer unsaturated oligosaccharides of up to 22 monosaccharide units (Figure 3C), the latter visible as distinct bands on agarose gels (Figure S2, lanes 2–4). This pattern suggests slower or less efficient depolymerization, with incomplete conversion of high‐molecular‐weight HA into ΔAN2. It should be noted that these comparisons of degradation speed are based on end‐point analyses of product profiles rather than direct kinetic measurements. While the accumulation of longer oligosaccharides in *B. acidifaciens* cultures is consistent with slower or less efficient HA depolymerization, this inference remains indirect. Definitive conclusions will require kinetic analyses that monitor the temporal dynamics of substrate breakdown and product formation. Such studies would provide a more quantitative framework for evaluating the efficiency and mechanistic differences of HA degradation between species. The production of specific HA degradation products (Figure 3) combined with sustained growth in HA‐based minimal medium provides strong evidence that both species can utilize HA as a carbon source, though with differing efficiencies. The production of longer HA‐derived oligosaccharides in *B. acidifaciens* cultures suggests partial degradation, a strategy commonly observed among *Bacteroides* species. This phenomenon also highlights the risk of inferring efficiency solely from endpoint product distribution (Zafar and Saier 2021).

Nevertheless, the observed product differences motivate several mechanistic hypotheses. *B. acidifaciens* possesses a nearly complete and highly conserved set of GAG‐specific PUL‐encoded proteins, with average amino acid identities > 82% compared with *B. thetaiotaomicron*, with the exception of surface proteins (BT3328–BT3330) (Table S3). Several explanations are possible. First, *B. acidifaciens* lacks experimentally validated data on the localization and regulation of its surface polysaccharide lyases (BT3328). In *B. thetaiotaomicron*, rapid HA depolymerization depends on BT3328 and associated binding proteins (BT3329–BT3330), which capture and cleave high‐molecular‐weight HA at the cell surface before periplasmic processing (Ndeh 2020). Even minor sequence differences in these proteins or differences in promoter architecture, expression timing, or regulatory control could slow substrate capture and transport. Second, the broader oligosaccharide profile in *B. acidifaciens* may reflect reduced periplasmic GH88 (BT3348) activity or altered SusC/D (BT3331 and BT3332) transporter specificity. Both protein families are known to undergo adaptive diversification in *Bacteroides* in response to niche‐specific glycan availability (Martens et al. 2009; Grondin et al. 2017), which can influence growth rate (Figure S1B,D). Finally, transcriptional regulation could differ between the two species. In *B. thetaiotaomicron*, GAG‐specific PUL‐encoded protein expression is strongly induced by HA, whereas in other *Bacteroides* species, constitutive or delayed induction has been observed (Cartmell et al. 2017).

Across all three strains, *B. acidifaciens*, *B. thetaiotaomicron*, and *P. heparinus*, unsaturated HA‐derived oligosaccharides were accompanied by saturated disaccharides (Figure 3). The presence of these saturated products is unexpected, as HA depolymerization by lyases proceeds via a β‐elimination mechanism that generates an unsaturated uronic acid at the nonreducing end (Sindelar et al. 2021; Ndeh 2020). Our observations are consistent with Pan et al. (2021), who reported that unsaturated disaccharides produced by lyase activity can undergo hydration of the C4–C5 double bond, yielding saturated products (Pan et al. 2021). Whether this conversion occurs spontaneously under culture conditions or is enzyme‐mediated remains unresolved. Possible explanations include nonenzymatic hydration influenced by pH, temperature, or redox conditions; activity of some hydrolases that accept unsaturated substrates; or culture‐dependent chemical modifications unrelated to canonical GAG catabolism. Discriminating among these possibilities, as enzymatic hydration would represent an unrecognized branch of microbial HA catabolism, whereas chemical hydration would reflect experimental artefacts. Targeted kinetic experiments, enzyme knockouts, and metabolite tracing will be needed to determine the biological relevance of saturated disaccharides.

In general, these results reinforce that pathway conservation does not necessarily equate to identical functional performance, underscoring the importance of direct phenotypic validation. To probe the molecular basis of these differences more deeply, we next performed a proteomic analysis of *B. acidifaciens* and *B. thetaiotaomicron* cultivated in HA‐based minimal medium versus standard nutrient‐rich medium, with the aim of identifying differential protein expression patterns that may underlie the observed variation in HA‐degradation efficiency.

### 2.4 Analysis of GAG‐Specific PUL‐Encoded Protein Expression Using Quantitative Proteomics

To investigate the GAG‐specific PUL‐encoded proteins, we cultivated *B. thetaiotaomicron* DSM 2079 and *B. acidifaciens* DSM 111135 in both standard medium and HA‐based minimal medium. Bacterial cells from each condition were harvested by centrifugation, washed, and processed for quantitative proteomic analysis. By comparing growth in a nutrient‐rich medium to a minimal medium with HA as the sole carbon source, we aimed to capture the full spectrum of proteomic changes, including both substrate‐specific machinery and broader metabolic adaptations required for utilizing this complex glycan.

**Figure 4:**
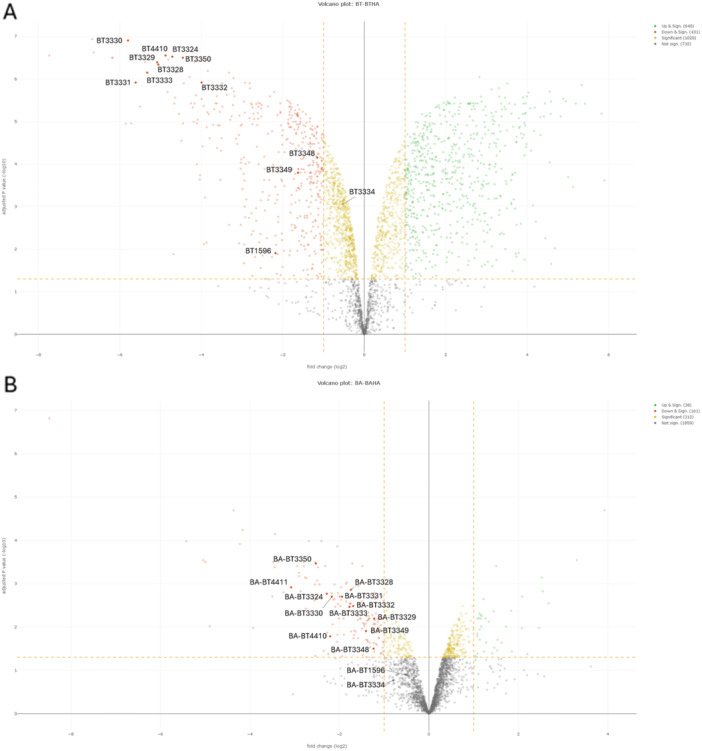
(A) Volcano plot of differential protein expression in *Bacteroides thetaiotaomicron* DSM 2079 cultured in standard medium (BT) versus hyaluronan (HA)‐based minimal medium (BTHA). Each point represents a detected protein, with log_2_ fold change on the *x*‐axis and –log_10_ adjusted *p* value on the *y*‐axis. Proteins significantly upregulated in the HA‐based medium (log_2_ FC ≥ 1, adjusted *p* ≤ 0.05) are shown in red, while proteins significantly upregulated under standard conditions (log_2_ FC ≤ − 1, adjusted *p* ≤ 0.05) are shown in green. Statistically significant proteins that do not meet the log_2_ fold‐change threshold are indicated in yellow, and nonsignificant proteins are shown in gray. Proteins involved in GAG metabolism are additionally highlighted and annotated in the plot. (B) Volcano plot of differential protein expression in *Bacteroides acidifaciens* DSM 111135 cultured in standard medium (BA) versus HA‐based minimal medium (BAHA). Each point represents a detected protein, with log_2_ fold change on the *x*‐axis and –log_10_ adjusted *p* value on the *y*‐axis. Proteins significantly upregulated in the HA‐based medium (log_2_ FC ≥ 1, adjusted *p* ≤ 0.05) are shown in red, while proteins significantly upregulated under standard conditions (log_2_ FC ≤ − 1, adjusted *p* ≤ 0.05) are shown in green. Statistically significant proteins that do not meet the log_2_ fold‐change threshold are indicated in yellow, and nonsignificant proteins are shown in gray. Proteins involved in GAG metabolism are additionally highlighted and annotated in the plot. GAG, glycosaminoglycan.

Across both conditions, quantitative proteomics identified 2873 proteins in *B. thetaiotaomicron* and 2397 proteins in *B. acidifaciens* (data deposited in ProteomeXchange, data set ID: PXD067466). In *B. thetaiotaomicron*, 640 proteins were significantly upregulated in standard medium and 431 in HA‐based minimal medium (Figure 4A), representing ~15% of the detected proteome. This reflects a substantial transcriptional and translational shift when HA is the sole carbon source. In *B. acidifaciens*, the expression profile was more constrained, with only 38 proteins upregulated in standard medium and 161 in HA‐based minimal medium (Figure 4B).

#### 2.4.1 Upregulation of Canonical GAG‐Specific PUL‐Encoded Proteins

Focusing on the GAG‐specific PUL‐encoding proteins previously defined by Ndeh (2020) in the *B. thetaiotaomicron* (Figure 1) (Ndeh 2020), we found that nearly the entire canonical GAG‐specific PUL‐encoded proteins were upregulated in *B. thetaiotaomicron* grown in HA‐based medium (Figure 4A). The only exception was BT3334, which, despite statistical significance, showed a fold change below 1. *B. acidifaciens* displayed a similar trend (Figure 4B), with most of the GAG‐specific PUL‐encoded proteins upregulated in HA‐based medium, except for sulfatases homologous to BT1596 (BA‐BT1596) and BT3334 (BA‐BT3334). As these sulfatases target sulfated GAGs, their absence is consistent with HA catabolism, which does not require desulfation (Cartmell et al. 2017).

#### 2.4.2 Upregulation of Non‐Canonical HA Degradation‐Associated Proteins

In addition to the canonical GAG‐specific PUL‐encoded proteins, non‐canonical HA degradation‐associated proteins also appear highly relevant. Notably, our analysis revealed a significant upregulation of BT4411, a DUF4627 domain‐containing protein, in *B. thetaiotaomicron* grown in HA‐based medium (Figure 4A). This protein is not part of the Ndeh (2020) canonical GAG‐specific PUL‐encoded protein set but has been proposed to participate in HA degradation based on genetic and functional studies (H. Liu et al. 2021). In *B. thetaiotaomicron*, BT4411 resides in PUL80 adjacent to BT4410 (Raghavan et al. 2014), a polysaccharide lyase from PL33 with high affinity to HA (Ndeh 2020). Previous data suggest BT4411 may function as a periplasmic accessory protein, possibly in substrate binding or enzyme complex organization, rather than as a hydrolase or lyase (H. Liu et al. 2021). It is induced by HA, which points to a specialized role in HA utilization (H. Liu et al. 2021; Overbeeke et al. 2022).

Strikingly, we also observed a significant upregulation of a BT4411 ortholog (BA‐BT4411) in *B. acidifaciens* (Figure 4B), accompanied by upregulation of its BT4410‐like neighbor. This is, to our knowledge, the first report linking BT4411 homologs to HA catabolism in *B. acidifaciens*. While *B. acidifaciens* clearly expresses canonical GAG‐specific PUL‐encoded proteins, the co‐induction of BT4410 and BT4411 orthologs (BA‐BT4410 and BA‐BT4411, respectively) suggests that it may also employ accessory components analogous to those in *B. thetaiotaomicron* PUL80. Thus, *B. thetaiotaomicron* and *B. acidifaciens* upregulate non‐canonical accessory proteins (BT4410/BT4411 homologs), suggesting a potentially conserved mechanism in *Bacteroides* species. The observation of similar upregulation patterns in two distinct *Bacteroides* species provides a strong foundation for hypothesizing a conserved mechanism across the genus, setting the stage for more comprehensive future studies.

#### 2.4.3 Functional Annotation and Metabolic Shifts

Functional classification of upregulated proteins by KEGG BlastKOALA revealed a decrease in carbohydrate metabolism proteins in *B. thetaiotaomicron* grown in HA‐based minimal medium, alongside a relative increase in proteins linked to amino acid metabolism and energy metabolism (Figure S3). This shift is consistent with reports showing that GAGs, including HA, serve as nutrient sources and induce broad transcriptional and metabolic responses in *Bacteroides* species (Cartmell et al. 2017; Koropatkin et al. 2012). Similar HA‐driven remodeling of CAZyme profiles has been reported in other *Bacteroides* species (Overbeeke et al. 2022; Martens et al. 2009). However, BlastKOALA annotated ~37% of proteins upregulated in standard medium and ~55% in HA‐based minimal medium. This limited coverage is typical for nonmodel anaerobes, where many proteins remain hypothetical or lineage‐specific (Kanehisa et al. 2016), and it constrains the depth of pathway reconstruction.

In *B. acidifaciens*, annotation rates for upregulated proteins were too low to support robust functional comparisons, precluding definitive conclusions about broader metabolic shifts under HA growth. Nevertheless, the induction of both canonical and non‐canonical GAG‐related proteins, including the BT4411 ortholog, provides new evidence that *B. acidifaciens* possesses a functional HA utilization system with accessory components not previously reported in this species.

### 2.5 Limitations of Our Study

This study combined large‐scale protein sequence analysis, targeted cultivation, and quantitative proteomics to evaluate whether *Bacteroides* species can be functionally grouped by their capacity to degrade HA. Our initial analysis followed the prevailing binary classification of *Bacteroides* species as HA degraders or non‐degraders. However, the observed inconsistencies suggest that this framework may oversimplify what is in fact a quantitative and context‐dependent phenotype. While this approach revealed the shortcomings of PL8/GH88‐based clustering, validated *B. acidifaciens* as a functional HA degrader, and identified the induction of both canonical and non‐canonical proteins during HA utilization, several limitations remain. First, the clustering relied mainly on PL8 and GH88 sequences, which proved insufficient to reliably distinguish HA‐degrading from non‐degrading species. Incorporating BT4410 and BT4411 as additional candidate markers could improve resolution, as both have been experimentally implicated in HA metabolism. However, their sparse annotation and limited representation in current databases currently constrain their broader application in comparative analyses. Second, the experimental design does not formally distinguish between protein expressions induced specifically by HA and general metabolic responses to nutrient‐limited conditions. Consequently, some proteins identified as upregulated may reflect nonspecific stress responses rather than direct roles in HA catabolism.

### 2.6 Future Perspectives

Future studies should aim to expand the comparative framework beyond PL8 and GH88 by incorporating BT4410/BT4411 homologs and other non‐canonical proteins into clustering strategies. Experimental validation across a broader panel of *Bacteroides* species and strains will be essential to capture the full diversity of HA‐degrading phenotypes and to disentangle cases where sequence conservation does not translate into functional activity. Targeted genetic approaches, such as knockouts and complementation assays, will be crucial to confirm the specific roles of BT4411 and its homologs. Additionally, biochemical characterization of degradation intermediates and accessory enzymes could reveal alternative routes of HA processing and clarify how these pathways contribute to overall GAG metabolism.

## 3 Conclusion

Our comparative clustering of PL8 and GH88 enzymes across *Bacteroides* species revealed that neither cluster membership nor high sequence identity to the *B. thetaiotaomicron* GAG‐specific PUL‐encoded proteins reliably predicts HA utilization. Experimental validation in *B. acidifaciens* confirmed that a species can harbor a nearly complete and highly conserved set of GAG‐specific PUL‐encoded proteins yet display slower and less complete depolymerization than the model degrader *B. thetaiotaomicron*, likely due to differences in surface polysaccharide lyases, transport systems, and regulatory mechanisms. Proteomic profiling demonstrated that *B. acidifaciens* upregulates both canonical GAG‐specific PUL‐encoded proteins and non‐canonical accessory proteins (BT4410/BT4411 homologs) during HA growth. The co‐induction of BT4410/BT4411 homologs with canonical GAG–PUL proteins makes them strong candidates for further investigation as potential biomarkers to improve predictive frameworks for HA utilization. Although our study is purely mechanistic, the identification of BT4410/BT4411 homologs associated with HA degradation has potential clinical relevance. Because HA is abundant in host tissues and widely used in therapeutics, variation in bacterial HA‐degrading capacity could influence mucosal barrier integrity, immune responses, and susceptibility to Inflammatory Bowel Disease and related disorders. Future work linking the presence and expression of these proteins to patient‐associated microbiota could clarify their contribution to gut health and disease risk.

## 4 Methods

### 4.1 Comparative Clustering of Enzymes Involved in GAG‐Degradation

Protein sequence data sets for enzymes from PL8 and GH88 were retrieved from UniProt (Bateman et al. 2021) and the NCBI database. Searches were performed using the terms “Polysaccharide lyase family 8” or “PL8” for PL8 enzymes, and “Glycoside hydrolase family 88,” “Unsaturated glucuronyl hydrolase,” “UGL,” or “GH88” for GH88 enzymes, with the organism filter set to *Bacteroides*. Fragmented and multispecies sequences were removed from the data sets.

Sequences were clustered using USEARCH (Edgar 2010) based on sequence distances, with match identity and optimal score set to 60% and all other parameters left at default values. The choice of the 60% identity threshold was based on empirical evaluation: thresholds from 40% to 80% were tested in increments of +10%, and 60% was selected as it provided a clustering resolution that avoided both excessive fragmentation and oversimplification, while producing heatmaps with visually distinct clustering patterns. The resulting clusters were processed with a custom Python script to generate binary matrices indicating the presence or absence of enzymes in each cluster for each *Bacteroides* species. Binary matrices were converted into distance matrices using the Jaccard index, which were then averaged and merged into a single distance matrix. This matrix was subjected to hierarchical clustering using Orange software (Demsar 2013). We evaluated clustering using only species with clear experimental labels. Species marked Mixed or NA were excluded from this analysis. We measured agreement between cluster assignments and these labels using the Adjusted Rand Index (ARI) and Normalized Mutual Information (NMI). As a simple prediction check, we gave each cluster the label that most of its labeled members had (majority vote) and then reported precision, recall, *F*1, and accuracy.

The resulting clusters were analyzed for identity of GAG‐specific PUL‐encoded proteins to those of *B. thetaiotaomicron*, used as a reference (Ndeh 2020). Reference sequences (BT1596, BT3324, BT3328–BT3334, BT3348–BT3350, and BT4410) were retrieved from UniProt and used as queries in BLASTp searches against the proteomes of *Bacteroides* species in each cluster.

### 4.2 Assessment of Conservation of the GAG‐Specific PUL‐Encoded Proteins in *B. acidifaciens*

To evaluate whether the GAG‐specific PUL‐encoded proteins are strain‐specific or conserved at the species level in *B. acidifaciens*, 13 reference proteins from the experimentally validated GAG degradation pathway of *B. thetaiotaomicron* (BT1596, BT3324, BT3328, BT3329, BT3330, BT3331, BT3332, BT3333, BT3334, BT3348, BT3349, BT3350, and BT4410) (Ndeh 2020) were used as queries in BLASTp searches against *B. acidifaciens* sequences in the NCBI database. Homologous sequences identified in this search were subsequently used as query sequences in a large‐scale BLASTp analysis against 230 predicted *B. acidifaciens* proteomes downloaded from NCBI. This second BLASTp search was performed locally in RStudio using the packages rBLAST, Biostrings, openxlsx, and dplyr. For each strain, only the top‐scoring hit per query was retained. Hits were filtered to include only those with ≥ 60% sequence identity and ≥ 30% query coverage to exclude partial or truncated sequences. A binary presence/absence matrix was generated to record the distribution of homologs across strains. For each protein, the mean sequence identity and standard deviation were calculated to assess conservation. All results were manually reviewed and analyzed in Microsoft Excel to evaluate the completeness and sequence conservation of GAG‐specific PUL‐encoded proteins across *B. acidifaciens* strains.

### 4.3 Bacterial Strains and Cultivation Conditions

*B. thetaiotaomicron* DSM 2079, *B. acidifaciens* DSM 111135, and *P. heparinus* DSM 2366 were obtained from the German Collection of Microorganisms and Cell Cultures (DSMZ). *B. thetaiotaomicron* DSM 2079 and *B. acidifaciens* DSM 111135 were cultivated anaerobically at 37°C in a Vinyl Coy Anaerobic Chamber (Coy Laboratory Products) maintained with a gas mixture of 85% N_2_, 10% CO_2_, and 5% H_2_. *B. thetaiotaomicron* DSM 2079 was grown for 24 h in Fastidious Anaerobic Broth (FAB; Neogen), while *B. acidifaciens* DSM 111135 was grown for 7 days in modified Gifu Anaerobic Medium Broth (mGAM; Himedia). *P. heparinus* DSM 2366 was cultivated aerobically at 28°C in Nutrient Medium (NM; 5 g/L peptone, 3 g/L meat extract; pH 7) for 5 days. Bacterial growth was monitored by measuring optical density at 600 nm (OD_600_) in 96‐well plates using an Infinite M200 iControl plate reader (Tecan). Culture purity was confirmed by three successive subcultures from single colonies on the respective agar media (FAB agar, mGAM agar, or NM agar; 1.5% agar; VWR). Final glycerol stocks (25% v/v) were prepared and stored at −80°C. Growth data were analyzed using GraphPad Prism 8 (GraphPad Software).

### 4.4 Cultivation of Bacterial Strains in HA‐Based Minimal Medium

*B. thetaiotaomicron* DSM 2079 and *B. acidifaciens* DSM 111135 were cultivated anaerobically at 37°C in HA‐based minimal medium containing high‐molecular‐weight HA (1500 kDa; sterile; Contipro) for 3 and 14 days, respectively, when bacterial strains reached the stationary phase. *P. heparinus* DSM 2366 was cultivated aerobically at 28°C for 7 days in the same medium. The HA‐based minimal medium contained (per liter): 1 g (NH_4_)_2_SO_4_, 2.26 g KH_2_PO_4_, 0.9 g K₂HPO_4_, 0.9 g NaCl, 0.0025 g hemin, 0.4 g cysteine HCl, 0.005 mL vitamin K_1_, 0.005 mg vitamin B_12_, 1.25 g HA (1500 kDa, sterile; Contipro), and 2 mL of a trace element solution containing FeSO_4_, CaCl_2_·2H_2_O, MgCl_2_·6H_2_O, and MnCl_2_·4H_2_O. Bacterial growth was monitored spectrophotometrically by measuring optical density at 600 nm (OD_600_) in 96‐well plates using an Infinite M200 iControl plate reader (Tecan). Growth data were analyzed using GraphPad Prism 8 (GraphPad Software).

### 4.5 Oligosaccharide Analysis by LC–MS

Following cultivation in HA‐based minimal medium, cultures were centrifuged at 6000*g* for 20 min at 4°C using a Centrifuge 5417 (Eppendorf) to separate bacterial cells from the supernatant. Supernatants from *B. thetaiotaomicron* DSM 2079, *B. acidifaciens* DSM 111135 (each grown in triplicate), and *P. heparinus* DSM 2366 were analyzed for HA degradation and the presence of HA‐derived oligosaccharides. Oligosaccharides were extracted with 400 μL of chilled (−80°C) methanol, and the resulting extracts were dissolved in 100 μL of 2 mmol/L ammonium acetate (pH 5.0). Chromatographic separation was performed on an Atlantis Premier BEH C18 AX column (50 × 2.1 mm, 1.7 μm; Waters) maintained at 60°C, using 2 mmol/L ammonium acetate (pH 5.0) and acetonitrile as mobile phases. The flow rate was 0.4 mL/min with the following acetonitrile gradient: 5% from 0 to 0.5 min, 5%–70% from 0.5 to 5.0 min, 70% from 5.0 to 6.0 min, 70%–5% from 6.0 to 6.3 min, and 5% from 6.3 to 8.0 min. HA‐derived oligosaccharides were analyzed using an Agilent 1290 Infinity II system coupled to a Agilent 6495 mass spectrometer equipped with an electrospray ionization source. Typical fragmentations involved the formation of glucuronic acid residues (saturated or unsaturated, depending on oligosaccharide type) and cleavage of *N*‐acetylglucosamine from the reducing end of the oligosaccharide. Identification was based on retention time and the presence of both characteristic transitions.

### 4.6 Detection of HA‐Derived Oligosaccharides by Agarose Gel Electrophoresis

Following cultivation in HA‐based minimal medium, bacterial cultures were centrifuged at 6000*g* for 20 min at 4°C using a Centrifuge 5417 (Eppendorf) to separate cells from the supernatant. Supernatants from *B. thetaiotaomicron* DSM 2079 and *B. acidifaciens* DSM 111135 (each grown in triplicate) were analyzed for HA‐derived oligosaccharides using agarose gel electrophoresis. As positive control, the supernatant from *P. heparinus* DSM 2366, cultivated aerobically in HA‐based minimal medium for 5 days at 28°C, was used. Sterile HA‐based minimal medium without inoculation served as a negative control. For electrophoresis, 12 µL of each supernatant was mixed with 3 µL of loading dye (0.02% bromophenol blue) and applied to a 0.5% agarose gel (0.5 g agarose in 100 mL of 1× TAE buffer, pH 8.55; Biozym). Gels were prerun at 100 V for 1 h to remove impurities before sample loading. Electrophoresis was then conducted at 100 V for 1.5 h. Following separation, gels were equilibrated in 30% ethanol (v/v) for 1 h at room temperature, stained overnight in the dark with a working solution of Stains‐All (Sigma Aldrich; stock solution: 12.5 mg Stains‐All in 5 mL of 96% ethanol, diluted 1:400 in 30% ethanol), and destained under ambient light in distilled water until HA‐derived oligosaccharide bands became visible.

### 4.7 Whole‐Cell Proteomic Profiling of *Bacteroides*

Following incubation, bacterial cells of *B. thetaiotaomicron* DSM 2079 and *B. acidifaciens* DSM 111135 (grown in triplicate) were harvested by centrifugation at 6000*g* for 20 min at 4°C using Centrifuge 5417 (Eppendorf), washed twice with sterile phosphate‐buffered saline, and concentrated to a final volume of 200 μL. Aliquots of 50 μL were used for downstream proteomic analysis. Proteins extracted from bacterial cell pellets were subjected to filter‐aided sample preparation as described elsewhere (Wiśniewski et al. 2009). The resulting peptides were analyzed by liquid chromatography‐tandem mass spectrometry (LC–MS/MS) performed using UltiMate 3000 RSLCnano system (Thermo Fisher Scientific) online coupled with timsTOF Pro mass spectrometer (Bruker). See the supplemental material section for details on the full analyses and data evaluation (Supporting Information Methods 1.1).

To investigate functional changes in protein expression, upregulated proteins from *B. thetaiotaomicron* DSM 2079 and *B. acidifaciens* DSM 111135 cultured in standard medium versus HA‐based minimal medium were annotated using the BlastKOALA tool using the BlastKOALA webserver for KEGG orthology assignment (Kanehisa et al. 2016).

### 4.8 AI Tools

Artificial intelligence tools (ChatGPT) were used exclusively for language editing and stylistic refinement. All aspects of experimental design, data collection, analysis, interpretation, and figure preparation were conducted independently by the authors and constitute original work.

## Author Contributions

**Martin Sindelar:** writing the original draft, investigation, methodology, bioinformatic data curation. **Anna Kocurkova:** investigation, data curation. **Matej Simek:** investigation, data curation. **Pavel Roudnicky:** proteomic analysis, data curation. **Gabriela Ambrozova:** project administration, writing – review and editing. **Lukas Kubala:** funding acquisition, conceptualization, writing – review and editing. **Kristyna Turkova:** conceptualization, supervision, writing the original draft, writing – review and editing. All authors have read and approved the published version of the manuscript.

## Ethics Statement

The authors have nothing to report.

## Conflicts of Interest

None declared.

## Supporting information

supplement_Sindelar_R1.

Table_S2.

Table_S3.

## Data Availability

Mass spectrometry proteomics data were deposited to the ProteomeXchange Consortium via PRIDE (Perez‐Riverol et al. 2019) partner repository under data set identifier PXD067466 (Reviewer access details: Log in to the PRIDE website using the following details: Project accession, PXD067466; Token, Lpsh0rntDkJD). Alternatively, the reviewer can access the data set by logging in to the PRIDE website using the following account details: username reviewer_pxd067466@ebi.ac.uk and password OZi0EvARqBbD.
