# Supplementary material for: Revisiting Hyaluronan Catabolism in Bacteroides: Pathway Conservation, Overlooked Proteins, and Predictive Accuracy
Source: Microbiologyopen. 2026 Feb 5;15(1):e70227. doi: 10.1002/mbo3.70227 (PMC12877422; doi:10.1002/mbo3.70227)
Supplement: Supplementary file 1 — supplement_Sindelar_R1. [file MBO3-15-e70227-s002.docx]

**Supplementary material**

Revisiting hyaluronan catabolism in *Bacteroides*: pathway conservation, overlooked proteins, and predictive accuracy

Martin Sindelar^1, 2^, Anna Kocurkova^1^, Matej Simek^3^, Pavel Roudnicky^4^, Gabriela Ambrozova^1^, Lukas Kubala^1, 2^, Kristyna Turkova^1, 2^***

*^1^ Institute of Biophysics of the Czech Academy of Sciences, Brno, Czech Republic*

*^2^ Institute of Experimental Biology, Faculty of Science, Masaryk University, Brno, Czech Republic*

*^3^ Contipro a.s., Dolní Dobrouč, Czech Republic*

*^4^* *Central European Institute of Technology (CEITEC), Masaryk University, Brno, Czech Republic*

**Correspondence: turkova@ibp.cz*

1. Supplementary Methods

1.1. Proteomic analysis of bacterial cells

Proteins from bacterial cell pellets were extracted in SDT buffer (4% SDS, 0.1M DTT, 0.1M Tris/HCl, pH 7.6) in a thermomixer (Eppendorf ThermoMixer C, 15 min, 95°C, 750 rpm). After that, all samples were centrifuged (15 min, 20,000 x g) and the supernatants (aprox. 70 μg of total protein) were used for filter-aided sample preparation (FASP) as described elsewhere [1] using 0.75 μg of trypsin (sequencing grade; Sigma-Aldrich). The resulting peptides were extracted into LC-MS vials by 2.5% formic acid (FA) in 50% acetonitrile (ACN) and 100% ACN with the addition of polyethylene glycol (final concentration 0.001%)[2] and concentrated in a SpeedVac concentrator (Thermo Fisher Scientific).

LC-MS/MS analyses of all peptide mixtures were done using UltiMate 3000 RSLCnano system (Thermo Fisher Scientific) connected to timsTOF Pro mass spectrometer (Bruker). Before LC separation, tryptic digests were online concentrated and desalted using a trapping column (Acclaim PepMap 100 C18, dimensions 300 μm ID, 5 mm long, 5 μm particles, Thermo Fisher Scientific). The trap column was then washed with 0.1% TFA and the peptides were eluted in backflush mode from the trapping column onto an analytical column (Aurora C18, 75μm ID, 250 mm long, 1.7 μm particles, Ion Opticks) by 90 min gradient program (flow rate 150 nl.min^-1^, 3-42% of mobile phase B; mobile phase A: 0.1% FA in water; mobile phase B: 0.1% FA in 80% ACN) followed by a system wash using 80% of mobile phase B. Equilibration of the trapping column and the analytical column was done before sample injection to sample loop. The analytical column was installed in the Captive Spray ion source (Bruker; temperatures set to 50 ºC) according to the manufacturer's instructions. Spray voltage and sheath gas were set to 1.4kV and 1, respectively.

MSn data were acquired in data-independent acquisition (DIA) mode with base method m/z range of 100-1700 and 1/k0 range of 0.6-1.4 V×s×cm^-2^. Enclosed DIAparameters.txt file defined m/z 400-1000 precursor range with equal windows size of 26 Th using two steps for each PASEF scan and cycle time of 100ms locked to 100% duty cycle**.** High sensitivity detection mode was used for the measurement of the vesicles samples to compensate the low protein digest input.

DIA data were evaluated separately for *B. acidifaciens* and *B. thetaiotaomicron*. Processing was done in DIA-NN (version 1.9.2)[3] in library-free mode against the cRAP database (based on [http://www.thegpm.org/crap](http://www.thegpm.org/crap/); 111 sequences in total, version 2018-11) and UniProtKB protein database for *B. acidifaciens* (based on <https://www.uniprot.org/proteomes/UP000491181>; version 2025-03, number of protein sequences: 4,156) and database for *B. thetaiotaomicron* (based on <https://www.uniprot.org/proteomes/UP000500882>; version 2025-03, number of protein sequences: 4,786). No optional, but carbamidomethylation as fixed modification and trypsin/P enzyme with 1 allowed missed cleavages and peptide length 7-30 were set during the library preparation. False discovery rate (FDR) control was set to 1% FDR. MS1 and MS2 accuracies as well as scan window parameters were set based on the initial test searches (median value from all samples ascertained parameter values). MBR was switched on.

Protein MaxLFQ intensities reported in the DIA-NN main report file were further processed using the software container environment (<https://github.com/OmicsWorkflows>), version 4.7.7a. Processing workflow is available upon request. Briefly, it covered: a) removal of low-quality precursors and contaminant protein groups, b) precursor intensities normalization by loessF algorithm and missing values imputation c) protein group MaxLFQ intensities calculation and log2 transformation, and d) differential expression analysis using LIMMA statistical test – proteins with adjusted p-value <0.05 and fold change >1 were considered as significantly changing.

Mass spectrometry proteomics data were deposited to the ProteomeXchange Consortium via PRIDE[4] partner repository under dataset identifier PXD067466 (Reviewer access details:Log in to the PRIDE website using the following details: Project accession: PXD067466 Token: Lpsh0rntDkJD, Alternatively, reviewer can access the dataset by logging in to the PRIDE website using the following account details:  Username: [reviewer_pxd067466@ebi.ac.uk](mailto:reviewer_pxd067466@ebi.ac.uk)  Password: OZi0EvARqBbD.


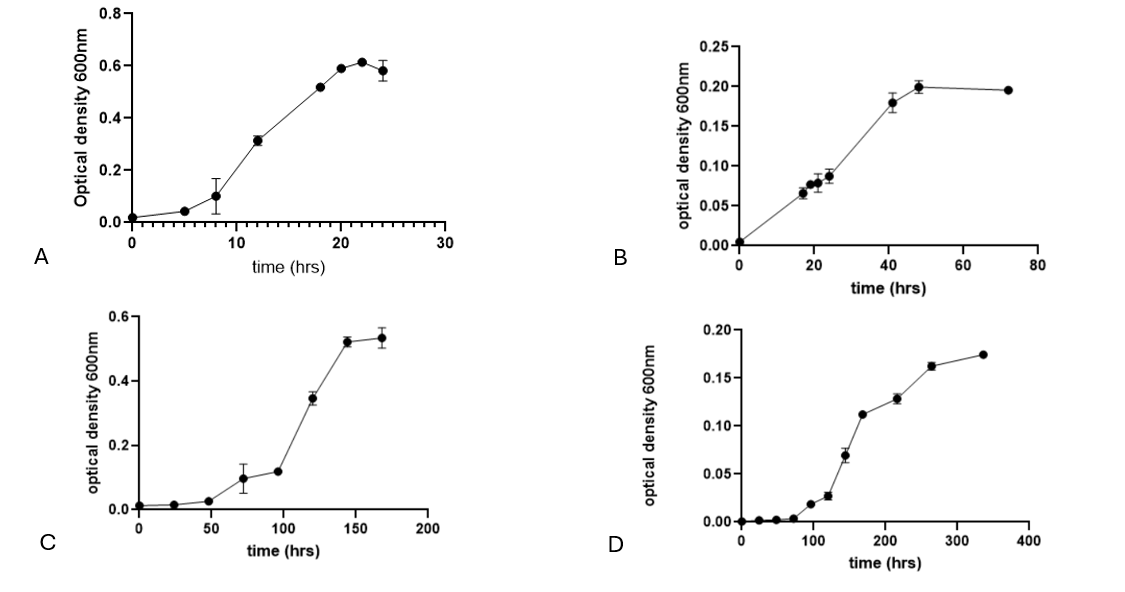


*Figure S1: Growth curves of Bacteroides thetaiotaomicron DSM2079 and Bacteroides acidifaciens DSM111135 cultured at 37 °C under anaerobic conditions in standard and HA-based minimal media. (A) B. thetaiotaomicron growth in standard Fastidious Anaerobic Broth; (B) B. thetaiotaomicron growth in HA-based minimal medium; (C) B. acidifaciens growth in standard modified Gifu Anaerobic Medium Broth; (D) B. acidifaciens growth in HA-based minimal medium. Bacterial growth was monitored spectrophotometrically by measuring optical density at 600 nm.* *Data represent mean ± SD (n = 3).*


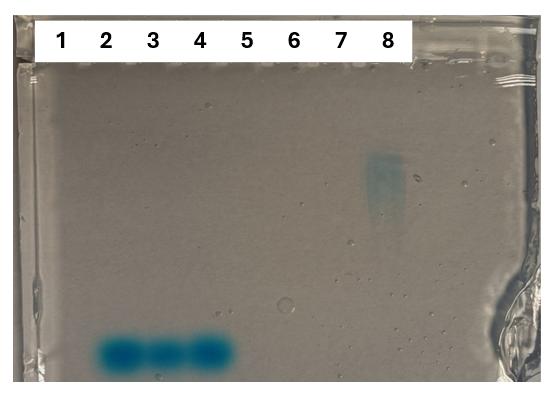


*Figure S2:* *Agarose gel electrophoresis of HA-based minimal medium following bacterial cultivation. Lane 1: medium after growth of Pedobacter heparinus DSM 2366; lanes 2–4: medium after growth of Bacteroides acidifaciens DSM 111135; lanes 5–7: medium after growth of Bacteroides thetaiotaomicron DSM 2079; lane 8: uninoculated HA-based minimal medium.*


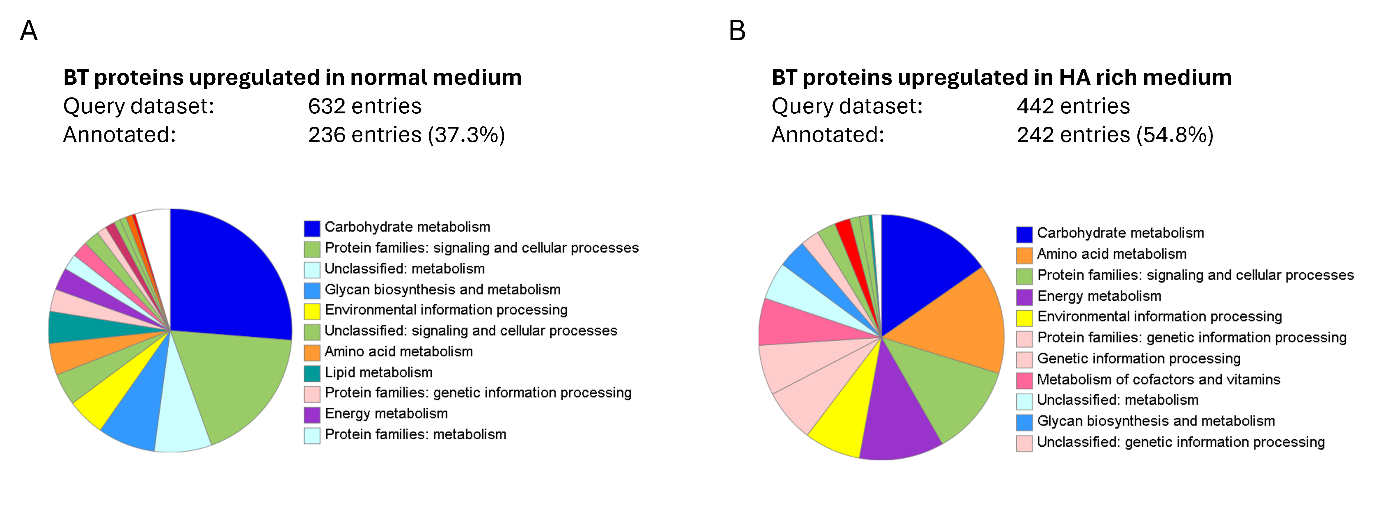


*Figure S3: KEGG functional classification of significantly upregulated proteins in Bacteroides thetaiotaomicron DSM2079 based on culture condition. Functional categories were assigned using BlastKOALA annotation [5]. (A) Proteins upregulated in standard medium; (B) Proteins upregulated in HA-based minimal medium.*

*Table S1****:*** *Summary of literature-based evidence on the ability of Bacteroides species to grow on hyaluronan as a sole carbon source. Green indicates confirmed growth, red indicates no growth, yellow represents conflicting reports, and gray denotes absence of published data. BT B.thetaiotaomicron, BH B. heparinolyticus, BZ B. zoogloeoformans, BC B. caecigallinarum, BCe B. cellulolyticus, BN B. neonati, BP B. pyogenes, BHe B. helcogenes, BNd B. ndongoniae, BTo B. togonis, BR B. rhinocerotis, BRo B. rodentium, BF B. fragilis, BHo B. hominis , BP B. propionicifaciens, BV B. vulgatus, BM B. muris, BMe B. mediterraneensis, BCo B. coprosuis, BI B. ilei, BNo B. nordii, BS B. salyersiae, BCu B. cutis, BG B. gallinaceum, BTi B. timonensis, BCel B. cellulosilyticus, BIn B. intestinalis, BO B. oleiciplenus, BSt B. stercorirosoris, BIh B. ihuae, BL B. luti, BE B. eggerthii, BU B. uniformis, BCl B. clarus, BSte B. stercoris, BFl B. fluxus, BGa B. gallinarum, BGr B. graminisolvens, BFa B. faecichinchillae, BB B. bouchesdurhonensis, BFae B. faecalis, BRe B. reticulotermitis, BZh B. zhangwenhongii, BD B. difficilis, BFi B. finegoldii, BFaec B. faecium, BCa B. cacae, BCon B. congonensis, BFaeci B. faecis, BCae B. caecimuris, BA B. acidifaciens, BX B. xylanisolvens, BOv B. ovatus*

|  | C1 | | | | | | | | | C2 | | C3 | | C4 | | | | | | | | | | C5 | | | | |
| --- | --- | --- | --- | --- | --- | --- | --- | --- | --- | --- | --- | --- | --- | --- | --- | --- | --- | --- | --- | --- | --- | --- | --- | --- | --- | --- | --- | --- |
|  | *BH* | *BZ* | *BC* | *BCe* | *BN* | *BP* | *BHe* | *BNd* | *BTo* | *BR* | *BRo* | *BF* | *BHo* | *BP* | *BV* | *BM* | *BMe* | *BCo* | *BI* | *BNo* | *BS* | *BCu* | *BG* | *BTi* | *BCel* | *BIn* | *BO* | *BSt* |
| [6] |  |  |  |  |  |  |  |  |  |  |  |  |  |  |  |  |  |  |  |  |  |  |  |  |  |  |  |  |
| [7] |  |  |  |  |  |  |  |  |  |  |  |  |  |  |  |  |  |  |  |  |  |  |  |  |  |  |  |  |
| [8] |  |  |  |  |  |  |  |  |  |  |  |  |  |  |  |  |  |  |  |  |  |  |  |  |  |  |  |  |
| [9] |  |  |  |  |  |  |  |  |  |  |  |  |  |  |  |  |  |  |  |  |  |  |  |  |  |  |  |  |
| [10] |  |  |  |  |  |  |  |  |  |  |  |  |  |  |  |  |  |  |  |  |  |  |  |  |  |  |  |  |
| [11] |  |  |  |  |  |  |  |  |  |  |  |  |  |  |  |  |  |  |  |  |  |  |  |  |  |  |  |  |
| [12] |  |  |  |  |  |  |  |  |  |  |  |  |  |  |  |  |  |  |  |  |  |  |  |  |  |  |  |  |
| Summary |  |  |  |  |  |  |  |  |  |  |  |  |  |  |  |  |  |  |  |  |  |  |  |  |  |  |  |  |

|  | C6 | | C7 | | | | | | | C8 | | | C9 | | | | C10 | | | | | | | | |
| --- | --- | --- | --- | --- | --- | --- | --- | --- | --- | --- | --- | --- | --- | --- | --- | --- | --- | --- | --- | --- | --- | --- | --- | --- | --- |
|  | *BIh* | *BL* | *BE* | *BU* | *BCl* | *BSte* | *BFl* | *BGa* | *BGr* | *BFa* | *BB* | *BFae* | *BRe* | *BZh* | *BD* | *BFi* | *BFaec* | *BCa* | *BCon* | *BFaeci* | *BCae* | *BA* | *BX* | *BOv* | BT |
| [6] |  |  |  |  |  |  |  |  |  |  |  |  |  |  |  |  |  |  |  |  |  |  |  |  |  |
| [7] |  |  |  |  |  |  |  |  |  |  |  |  |  |  |  |  |  |  |  |  |  |  |  |  |  |
| [8] |  |  |  |  |  |  |  |  |  |  |  |  |  |  |  |  |  |  |  |  |  |  |  |  |  |
| [9] |  |  |  |  |  |  |  |  |  |  |  |  |  |  |  |  |  |  |  |  |  |  |  |  |  |
| [10] |  |  |  |  |  |  |  |  |  |  |  |  |  |  |  |  |  |  |  |  |  |  |  |  |  |
| [11] |  |  |  |  |  |  |  |  |  |  |  |  |  |  |  |  |  |  |  |  |  |  |  |  |  |
| [12] |  |  |  |  |  |  |  |  |  |  |  |  |  |  |  |  |  |  |  |  |  |  |  |  |  |
| Summary |  |  |  |  |  |  |  |  |  |  |  |  |  |  |  |  |  |  |  |  |  |  |  |  |  |

Supplementary references

1. Wisniewski, J.R., et al., *Universal sample preparation method for proteome analysis.* Nature Methods, 2009. **6**(5): p. 359-U60.

2. Stejskal, K., D. Potesil, and Z. Zdráhal, *Suppression of Peptide Sample Losses in Autosampler Vials.* Journal of Proteome Research, 2013. **12**(6): p. 3057-3062.

3. Demichev, V., et al., *DIA-NN: neural networks and interference correction enable deep proteome coverage in high throughput.* Nature Methods, 2020. **17**(1): p. 41-+.

4. Perez-Riverol, Y., et al., *The PRIDE database and related tools and resources in 2019: improving support for quantification data.* Nucleic Acids Research, 2019. **47**(D1): p. D442-D450.

5. Kanehisa, M., Y. Sato, and K. Morishima, *BlastKOALA and GhostKOALA: KEGG Tools for Functional Characterization of Genome and Metagenome Sequences.* Journal of Molecular Biology, 2016. **428**(4): p. 726-731.

6. Fang, Z.Y., et al., *Degradation and fermentation of hyaluronic acid by Bacteroides spp. from the human gut microbiota.* Carbohydrate Polymers, 2024. **334**.

7. Pan, L., et al., *In vitro fermentation of hyaluronan by human gut microbiota: Changes in microbiota community and potential degradation mechanism.* Carbohydrate Polymers, 2021. **269**.

8. Overbeeke, A., et al., *Nutrient niche specificity for glycosaminoglycans is reflected in polysaccharide utilization locus architecture of gut Bacteroides species.* Frontiers in Microbiology, 2022. **13**.

9. Akazawa, H., et al., *Isolation and identification of hyaluronan-degrading bacteria from Japanese fecal microbiota.* Plos One, 2023. **18**(5).

10. Simek, M., et al., *Molecular weight and gut microbiota determine the bioavailability of orally administered hyaluronic acid.* Carbohydrate Polymers, 2023. **313**.

11. Ndeh, D., et al., *Metabolism of multiple glycosaminoglycans by Bacteroides thatiotaomicron is orchestrated by a versatile core genetic locus* Nature Communications, 2020. **11**(1).

12. Kawai, K., et al., *Probiotics in human gut microbiota can degrade host glycosaminoglycans.* Scientific Reports, 2018. **8**.
